# The Induced Immune Response in Patients With Infectious Spondylodiscitis: A Prospective Observational Cohort Study

**DOI:** 10.3389/fimmu.2022.858934

**Published:** 2022-03-14

**Authors:** Josefine Amalie Loft, Dina Leth Møller, Rebekka Faber Thudium, Jenny Dahl Knudsen, Sisse Rye Ostrowski, Åse Bengård Andersen, Susanne Dam Nielsen

**Affiliations:** ^1^ Viro-Immunology Research Unit, Department of Infectious Diseases, Copenhagen University Hospital, Rigshospitalet, Copenhagen, Denmark; ^2^ Department of Clinical Microbiology, Copenhagen University Hospital, Rigshospitalet, Copenhagen, Denmark; ^3^ Department of Clinical Immunology, Copenhagen University Hospital, Rigshospitalet, Copenhagen, Denmark; ^4^ Department of Clinical Medicine, Faculty of Health and Medical Sciences, University of Copenhagen, Copenhagen, Denmark; ^5^ Department of Infectious Diseases, Copenhagen University Hospital, Rigshospitalet, Copenhagen, Denmark; ^6^ Department of Surgical Gastroenterology and Transplantation, Copenhagen University Hospital, Rigshospitalet, Copenhagen, Denmark

**Keywords:** TruCulture^®^, induced immune response, *Staphylococcus aureus*, whole blood assay, immunologic profiling, immune deficiency, spondylodiscitis

## Abstract

**Introduction:**

Infectious spondylodiscitis is a rare infection of the intervertebral disc and the adjacent vertebral bodies that often disseminates and requires long-term antibiotic therapy. Immunologic profiling of patients with infectious spondylodiscitis could allow for a personalized medicine strategy. We aimed to examine the induced immune response in patients with infectious spondylodiscitis during and after antibiotic therapy. Furthermore, we explored potential differences in the induced immune response depending on the causative pathogen and the dissemination of the disease.

**Methods:**

This was a prospective observational cohort study that enrolled patients with infectious spondylodiscitis between February 2018 and August 2020. A blood sample was collected at baseline, after four to six weeks of antibiotic therapy (during antibiotic therapy), and three to seven months after end of antibiotic therapy (post-infection). The induced immune response was assessed using the standardized functional immune assay TruCulture^®^. We used a panel of three immune cell stimuli (lipopolysaccharide, Resiquimod and polyinosinic:polycytodylic acid) and an unstimulated control. For each stimulus, the induced immune response was assessed by measuring the released concentration of Interleukin (IL)-1β, IL-6, IL-8, IL-10, IL-12p40, IL-17A, Interferon-γ (IFN-γ) and Tumor necrosis factor-α (TNF-α) in pg/mL.

**Results:**

In total, 49 patients with infectious spondylodiscitis were included. The induced immune responses were generally lower than references at baseline, but the cytokine release increased in patients after treatment with antibiotic therapy. Post-infection, most of the released cytokine concentrations were within the reference range. No significant differences in the induced immune responses based on stratification according to the causative pathogen or dissemination of disease were found.

**Conclusion:**

We found lower induced immune responses in patients with infectious spondylodiscitis at baseline. However, post-infection, the immune function normalized, indicating that an underlying immune deficiency is not a prominent factor for spondylodiscitis. We did not find evidence to support the use of induced immune responses as a tool for prediction of the causative pathogen or disease dissemination, and other methods should be explored to guide optimal treatment of patients with infectious spondylodiscitis.

## Introduction

Infectious spondylodiscitis is a rare infection of the intervertebral disc and the adjacent vertebral bodies that can disseminate to multiple foci, including the central nervous system. Infectious spondylodiscitis is often severe and associated with morbidity and mortality, up to 68% of the patients develop abscesses, and 65% experience sequelae (1). Disseminated spondylodiscitis is often complicated, and according to Danish Society of Infectious Diseases guidelines, the recommended duration of antibiotic therapy is six weeks in non-complicated and 12 weeks in complicated cases, respectively (2).

During recent decades, the incidence of infectious spondylodiscitis has increased, possibly due to an aging population with associated comorbidities, larger proportion of invasive procedures, and improved diagnostic imaging (3–6). Haematogenic spread of *Staphylococcus aureus (S. aureus)* is the main etiology of infectious spondylodiscitis (1–3, 5, 7). Empiric antibiotic therapy targets *S. aureus*, but up to 30% of cases are due to Gram-negative bacteria causing possible treatment failure (3, 4, 8). Despite extensive investigations with blood cultures and/or surgical biopsies, 10-30% of cases remain culture negative (3, 9). This underlines the relevance of early pathogen detection for optimal treatment, and different immune responses to these pathogens may be of clinical relevance.

The pathogenesis leading to infectious spondylodiscitis is incompletely understood, and the role of the immune system is largely unknown (10). To our knowledge, only one study has assessed the adaptive immune function in patients with infectious spondylodiscitis using a non-standardized method (10). However, standardized functional immune assays such as TruCulture^®^ which quantifies the immune response to different customized stimulations may provide additional information and suggest novel immunopathological mechanisms in infectious spondylodiscitis. Furthermore, direct measurements of the immune response in patients with infectious spondylodiscitis could potentially serve as a predictor of disseminated disease, allowing for a personalized medicine strategy.

In this prospective observational cohort study, we aimed to examine the induced immune response using the standardized functional immune assay TruCulture^®^ in patients with infectious spondylodiscitis during and after antibiotic therapy. Furthermore, we aimed to investigate potential differences in the immune response depending on the causative pathogen and dissemination of the disease.

First, we hypothesized that patients with infectious spondylodiscitis would elicit immune hyporesponsiveness during acute infection, indicating (potentially reversible) immune deficiency in patients with acute infectious spondylodiscitis. Second, we hypothesized that, post-infection, patients with infectious spondylodiscitis would elicit immune hyporesponsiveness compared to references, indicating an underlying immune deficiency in patients developing infectious spondylodiscitis. Third, we hypothesized that the induced immune response would differ based on the causative pathogen and dissemination of the disease.

## Materials and Methods

### Study Design and Population

This prospective observational cohort study included 49 patients diagnosed with infectious spondylodiscitis admitted to the Department of Infectious Diseases at Rigshospitalet, Copenhagen, Denmark between February 2018 and August 2020. Inclusion criteria were: Age ≥ 18 years, diagnosed with infectious spondylodiscitis and oral and written consent. Three blood samples were collected from each participating patient, at the earliest possible time after diagnosis (baseline), after four to six weeks of antibiotic therapy (during antibiotic therapy), and three to seven months after end of antibiotic therapy (post-infection). Thus, each patient was his/her own control. All blood samples were analyzed with TruCulture^®^, and we compared the induced as well as the unstimulated immune response of the same patients at timepoints with and without active disease.

The cytokine concentrations in the first blood sample (baseline) represented the immune response to ongoing infectious spondylodiscitis. The cytokine concentrations in the second blood sample (during antibiotic therapy) represented the immune response during antibiotic therapy. The cytokine concentrations in the third blood sample (post-infection) represented the patients’ habitual immune function, and this timepoint was selected to allow for immune reconstitution.

### Ethics

The study was approved by The Research Ethics Committee of the Capital Region of Denmark (VEK) (H-17024315, approval number 79291), and The Danish Data Protection Agency (RH-2016-47, I-suite 04433), and it was conducted in accordance with the Declaration of Helsinki.

### TruCulture^®^

TruCulture^®^ (Myriad, RBM, Austin, USA) is a standardized functional immune assay which determines the immune response after stimulation in whole blood by quantifying the release of cytokines and chemokines in the supernatant. In this study, the TruCulture^®^ assay consisted of three different immune stimulations, mimicking the presence of Gram-negative bacteria [bacterial endotoxin, lipopolysaccharide, LPS, from *Escherichia coli* (*E. coli*), cat#782-001261)], and two viral agents [single stranded RNA virus; Resiquimod, R848 (cat#782-001264) and double stranded RNA virus; polyinosinic:polycytodylic acid, PolyI:C (cat#782-001282)], respectively. These stimulants activate different immunologic signaling pathways, including Toll-Like Receptors (TLR), with LPS activating TLR4, R848 activating TLR7/8 and PolyI:C activating TLR3 (11, 12). Furthermore, one TruCulture^®^ media without any stimulant serves as an unstimulated control.

The method of TruCulture^®^ sampling has been reported in previous studies (13–16). In brief, peripheral blood was collected in lithium heparin anticoagulated whole blood tubes and transferred into the four prewarmed TruCulture^®^ tubes after 60 minutes (+/- 15 min). The TruCulture^®^ tubes were custom-made with reduced heparin due to the blood sampling in lithium heparin anticoagulated whole blood tubes (11). The tubes were then incubated for 22 hours (+/- 30 min) at 37°C in a digital dry block heater (WWR International A/S, Søborg, Denmark). The supernatant was subsequently harvested and stored at -20°C for 1-7 days, then transferred to -80°C until use. Bulk analysis of the cytokines was performed. Data output were the concentration of the following cytokines and chemokine reported in pg/mL: Interleukin (IL)-1β, IL-6, IL-8, IL-10, IL-12p40, IL-17A, Interferon- γ (IFN-γ) and Tumor necrosis factor-α (TNF-α), measured in the supernatant by an 8-plex Luminex (LX200, R&D Systems, BIO-Teche LTD) according to manufacturer’s recommendations. The TruCulture^®^ assays were performed in singlets. For each cytokine, a reference interval based on the 5-95% range of the cytokine concentration based on a healthy individuals reference was provided by the Department of Clinical Immunology, Rigshospitalet, Copenhagen.

### Clinical Characteristics and Definitions

Clinical data were collected from patient records and included demographics, comorbidities, medication, microbiology and biochemistry.

Infectious spondylodiscitis was defined as an acute or chronic infection of the vertebrae and intervertebral disc. Site of infection was registered according to the involved level(s) of the spine, as cervical and/or thoracal and/or lumbar and/or sacral. Infections of the cervicothoracic junction (C7/Th1), the thoracolumbar junction (Th12/L1), and the lumbosacral junction (L5/S1) were defined as cervical, thoracal, and lumbar site of infection, respectively.

Local disease was defined as an infection of one or more adjacent vertebrae and disci without dissemination to other foci. Disseminated disease was defined as infectious spondylodiscitis with more than one focus of infection, including meningitis, endocarditis, abscess formation (epidural and/or paravertebral and/or psoas), >1 vertebral focus (i.e. more than one vertebral focus separated by more than one uninfected discus or vertebrae) and other disseminated infections, including soft tissue accumulations.

The microbiological diagnosis of spondylodiscitis was based on minimum one of the following: Blood culture, open biopsy, percutaneous biopsy, abscess aspirate and/or lumbar puncture. The microbiological diagnosis was mainly done by routine aerobic and anaerobic cultures of biopsies and other samples, but also by 16S/18S polymerase chain reaction.

### Statistical Analyses

Data were reported as medians and interquartile ranges for continuous data, and frequency counts and percentages for categorical data. Exact Wilcoxon-Mann-Whitney Test was used for unpaired non-parametric continuous data, and Exact Wilcoxon-Pratt Signed-Rank Test for paired non-parametric continuous data (R-package *coin*). The statistical analyses were performed both as explorative analyses without correction for multiple comparisons as well as with correction for multiple comparisons using the Benjamini-Hochberg method. To adjust for zero inflation, we added 0.01 to all cytokine concentrations, and cytokine concentrations were transformed using the base-10 logarithmic scale when plotting the results. Adjusted p-values < 0.05 were considered statistically significant. Only results that remained statistically significant after correction for multiple comparisons were shown in the results section. All statistical analyses were performed in R-studio version 3.6.1 (R Foundation for Statistical Computing, Vienna, Austria).

## Results

### Study Population and Demographics

In total, 49 patients with infectious spondylodiscitis were included, of which 31 (63%) were male. The median age was 65 years (IQR 54-76). The majority of patients (73%) had disseminated disease, and 17 (32%) were infected with *S. aureus*. The median time from onset of symptoms to diagnosis was 25 days (IQR 13-94). Additional baseline characteristics are listed in **Table 1**.

**Table 1:** Baseline characteristics of patients with infectious spondylodiscitis.

Characteristics	N = 49
Male sex, n (%)	31 (63)
Age, median (IQR)	65 (54-76)
**Risk factors, n (%)**	
Active intravenous drug use	5 (10)
Alcohol use: >7 (females) or >14 (males) units per week	8 (16)
Immunosuppressive therapy	
Current	6 (12)
Former	7 (14)
Former spinal surgery	5 (10)
< 1 month prior to inclusion	1 (2)
**Disease manifestation, n (%)**	
Local disease	13 (27)
Disseminated disease (>1 focus)	36 (74)
Meningitis	3 (6)
Endocarditis	1 (2)
Epidural abscess	19 (39)
Paravertebral abscess	6 (12)
Psoas abscess	8 (16)
>1 vertebral focus	5 (10)
Other^a^	11 (22)
Days from onset of symptoms to inclusion, median (IQR)^b^	35 (16-93)
Days from onset of symptoms to diagnosis, median (IQR)	25 (13-94)
Days from onset of symptoms to start of antibiotic therapy, median (IQR)	24 (6-89)
**Microbiology, n (%)**	
*Staphylococcus aureus* (including MRSA n = 1)	17 (32)
Gram-negative bacteria^c^	6 (11)
Culture negative	10 (19)
Other^d^	21 (39)
Monomicrobial	45 (92)
Polymicrobial^e^	4 (8)

IQR, interquartile range; MRSA, methicillin resistant Staphylococcus aureus; N, number.

a Aortitis, pleural abscess, soft tissue affection, - component, - accumulation, soft tissue abscesses around the shoulder joint.

b 2/49 patients did not have data on debut of symptoms specified in their records.

c E. coli (n = 4), Proteus species (n = 1), Enterobacter cloacae (n = 1).

d Streptococcal species (n = 8), Gardnerella vaginalis (n = 1), Enterococci (n = 4), Coagulase-negative staphylococci (n = 2), Cutibacterium acnes (n = 4), Mycobacterium tuberculosis (n = 1), Solobacterium moorei (n = 1).

e S. aureus + Enterococcus faecium (n = 1) - S. aureus + Cutibacterium acnes + Coagulase-negative staphylococcus (n = 1) - S. aureus + Streptococcus dysgalactiae (n = 1) - E. coli + Enterococcus faecium (n = 1).

The median time from diagnosis to inclusion was 4 days (IQR 3-10). Additional information on collection of blood samples for TruCulture^®^ are listed in **Table 2**.

**Table 2:** Blood samples for TruCulture^®^.

Blood samples for TruCulture^®^	
First sample, n (%)	49 (100)
Second sample, n (%)	43 (88)
Third sample, n (%)	19 (39)
Days from start of antibiotic therapy to first sample, median (IQR)	5 (3-11)
Days from diagnosis to first sample, median (IQR)	4 (3-10)
Weeks from start of antibiotic therapy to second sample, median (IQR)	5 (4-7)
Months^a^ from end of antibiotic therapy to third sample, median (IQR)	4.7 (4.2-5.2)

IQR, interquartile range; N, number.

a Months from end of antibiotic therapy to third sample was calculated by dividing days with 30.4167.

### The Induced Immune Response in Patients With Infectious Spondylodiscitis and References

#### Stimulated Samples

**Figure 1:**
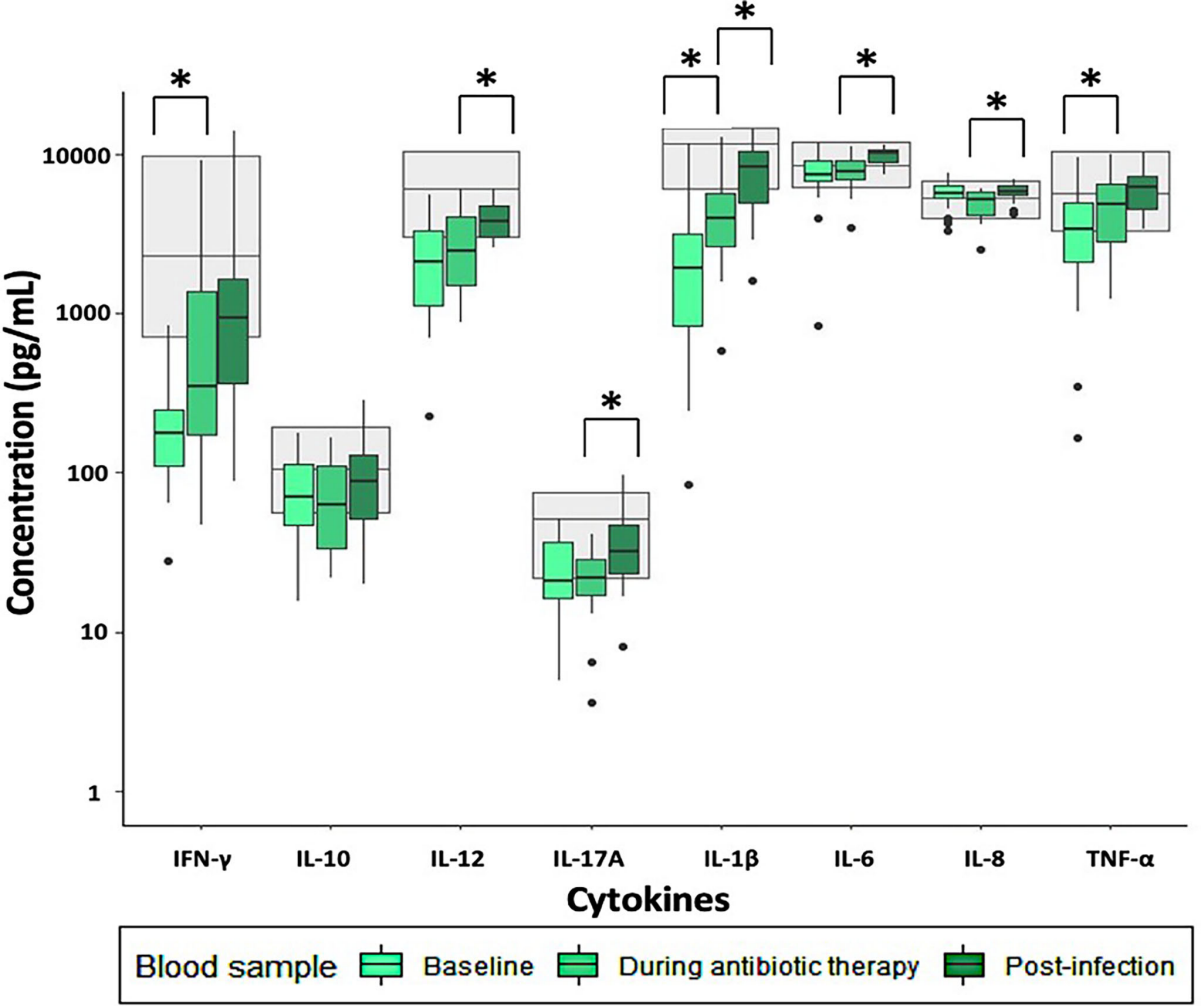
Immune response to LPS stimulation in patients with infectious spondylodiscitis at baseline, during antibiotic therapy and post-infection. The grey areas represent the 5-95% reference range of the healthy individuals. Cytokine concentrations were compared using Exact Wilcoxon-Pratt Signed-Rank Test for paired, non-parametric data. P-values < 0.05 after Benjamini-Hochberg correction were considered statistically significant. *P-value < 0.05.

**Figure 2:**
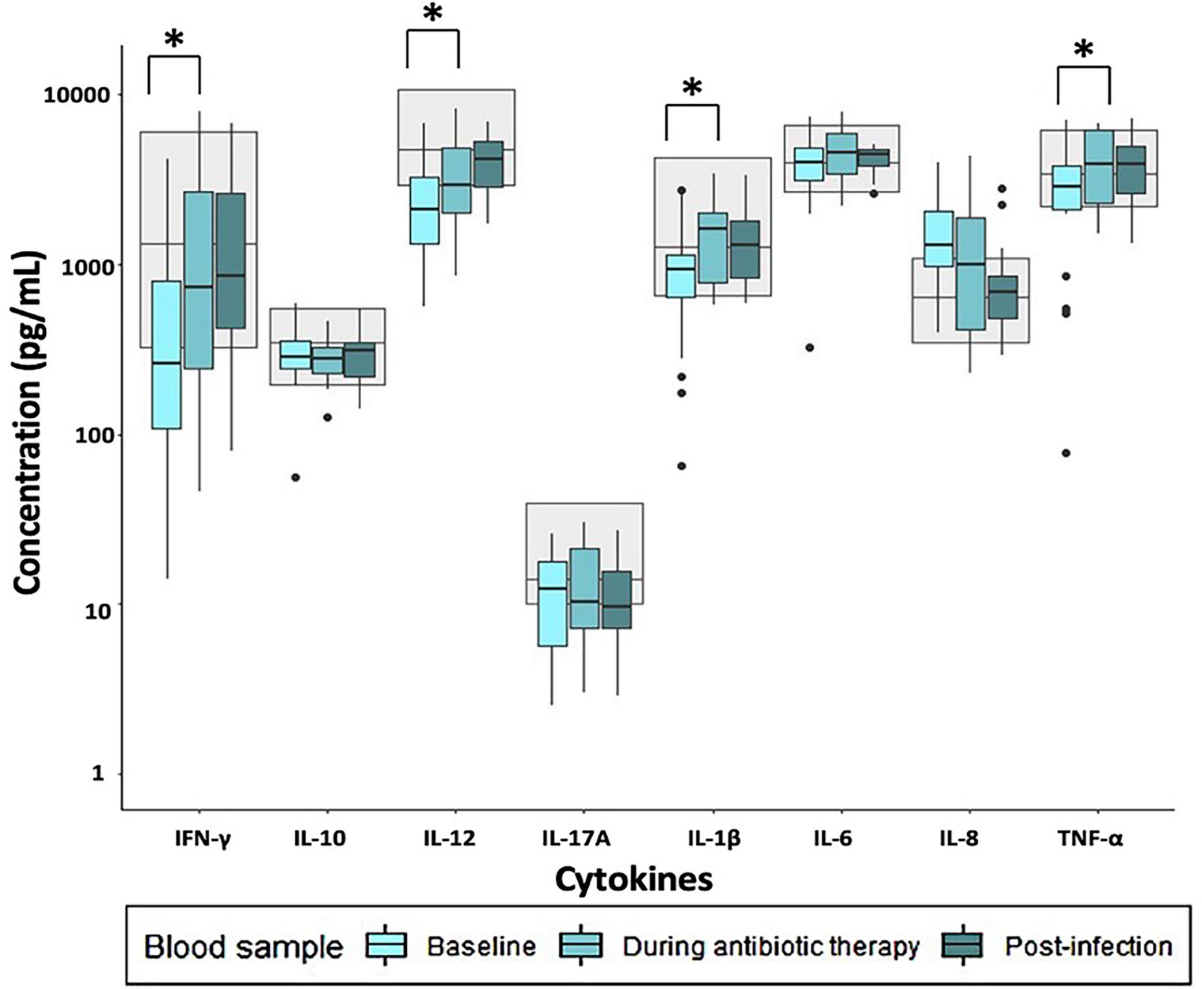
Immune response to R848 stimulation in patients with infectious spondylodiscitis at baseline, during antibiotic therapy and post-infection. The grey areas represent the 5-95% reference range of the healthy individuals. Cytokine concentrations were compared using Exact Wilcoxon-Pratt Signed-Rank Test for paired, non-parametric data. P-values < 0.05 after Benjamini-Hochberg correction were considered statistically significant. *P-value < 0.05.

**Figure 3:**
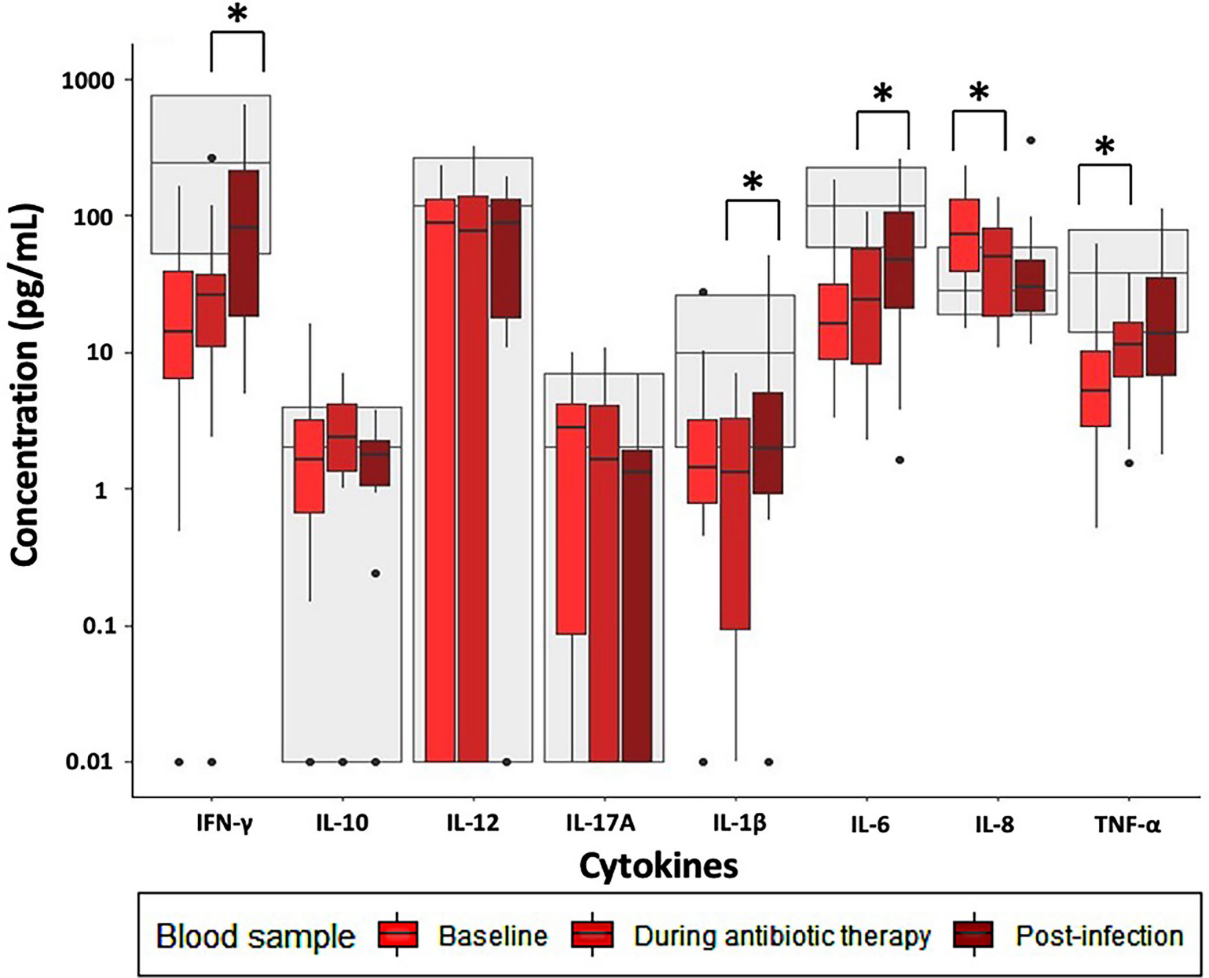
Immune response to Poly:IC stimulation in patients with infectious spondylodiscitis at baseline, during antibiotic therapy and post-infection. The grey areas represent the 5-95% reference range of the healthy individuals. Cytokine concentrations were compared using Exact Wilcoxon-Pratt Signed-Rank Test for paired, non-parametric data. P-values < 0.05 after Benjamini-Hochberg correction were considered statistically significant. *P-value < 0.05.

Most of the induced immune responses were either below or within the reference range at baseline and during antibiotic therapy (**Figures 1**–**3**).

Across all stimulations, TNF-α release was significantly lower at baseline compared to during antibiotic therapy (**Supplementary Table 1**). Furthermore, LPS- and R848 stimulated IFN-γ and IL-1β, and R848 stimulated IL-12 were significantly lower at baseline than during antibiotic therapy. However, Poly:IC stimulated IL-8 was significantly higher at baseline than during antibiotic therapy (**Supplementary Table 1**).

Post-infection, most of the induced immune responses were higher than at baseline and were within the reference range (**Figures 1**–**3**).

LPS stimulated IL-12, IL-17A, IL-1β, IL-6 and IL-8 were significantly higher post-infection than during antibiotic therapy, as well as Poly:IC stimulated IFN-γ, IL-1β and IL-6 (**Supplementary Table 2**). No other significant changes in the stimulated samples were observed when post-infection samples were compared to during antibiotic therapy.

#### Unstimulated Samples

**Figure 4:**
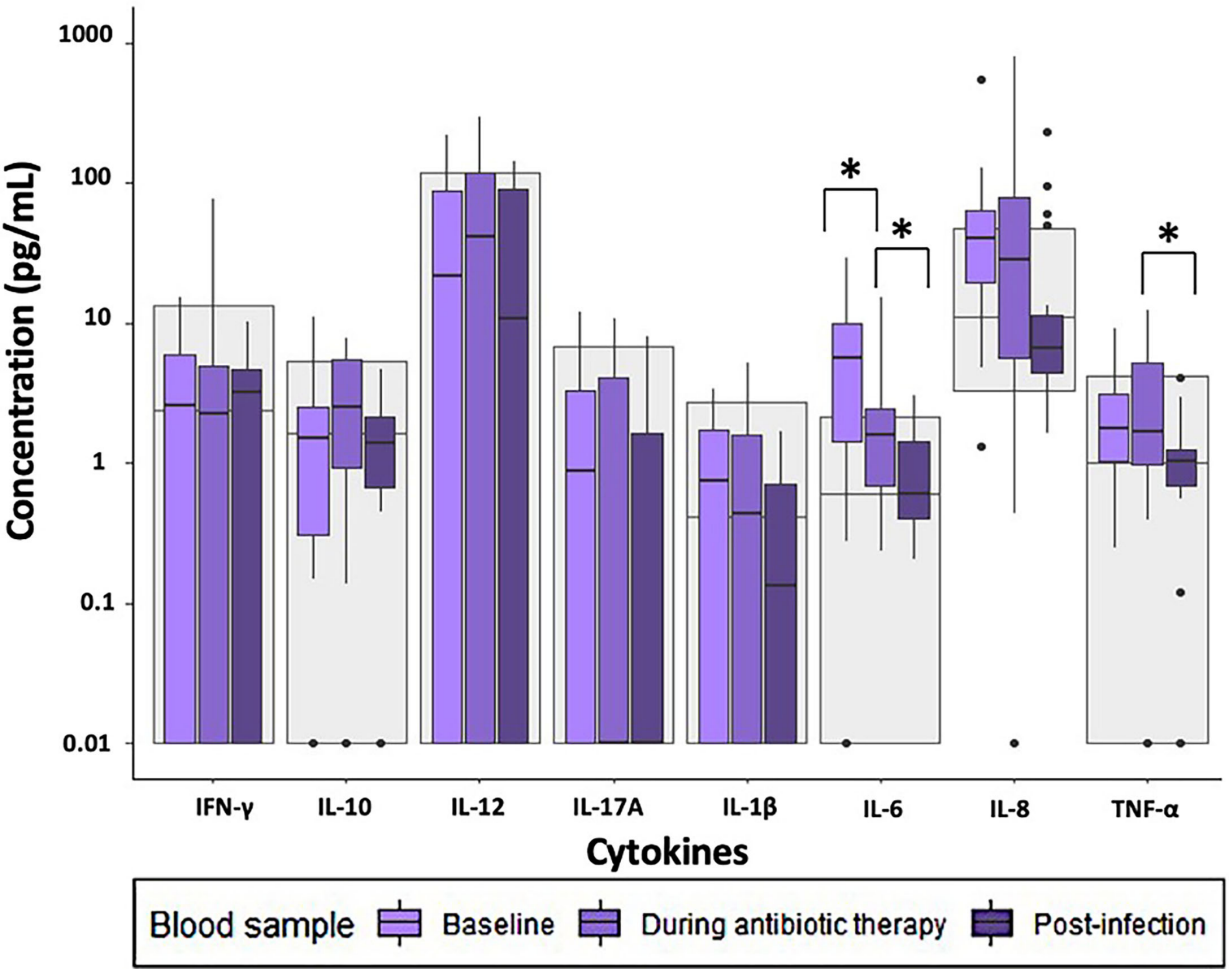
Cytokine concentrations in unstimulated samples in patients with infectious spondylodiscitis at baseline, during antibiotic therapy and post-infection. The grey areas represent the 5-95% reference range of the healthy individuals. Cytokine concentrations were compared using Exact Wilcoxon-Pratt Signed-Rank Test for paired, non-parametric data. P-values < 0.05 after Benjamini-Hochberg correction were considered statistically significant. *P-value < 0.05.

At baseline and during antibiotic therapy, most of the unstimulated cytokine concentrations in the patients with spondylodiscitis were within the reference range (**Figure 4**).

In the unstimulated samples, the concentration of IL-6 was significantly higher at baseline than during antibiotic therapy (**Supplementary Table 1**).

The unstimulated cytokine concentrations post-infection were within the reference range, and the concentrations of TNF-α and IL-6 were significantly lower post-infection than during antibiotic therapy (**Supplementary Table 2**). No other significant changes in cytokine concentrations were observed when comparing post-infection samples with those during antibiotic therapy.

### Comparison of the Induced Immune Response in Patients With Infectious Spondylodiscitis Caused by *S. aureus* Versus Gram-Negative Bacteria at Baseline

To determine differences in the induced immune response in patients with infectious spondylodiscitis caused by *S. aureus* (n=17) and infectious spondylodiscitis caused by Gram-negative bacteria (n=6), we compared all cytokine responses to all three stimuli and the unstimulated control in these two groups. There were no significant differences in any of the induced immune responses between patients with infectious spondylodiscitis caused by *S. aureus* and Gram-negative bacteria (**Supplementary Table 3**).

### Comparison of the Induced Immune Response in Patients With Infectious Spondylodiscitis With Local *Versus* Disseminated Disease at Baseline

To determine differences in the induced immune response in patients with infectious spondylodiscitis with local disease (n=13) and patients with infectious spondylodiscitis with disseminated disease (n=36), we compared all cytokine responses to all three stimuli and the unstimulated control in these two groups. There were no significant differences in any of the induced immune responses between infectious spondylodiscitis patients with local disease and disseminated disease (**Supplementary Table 4**).

## Discussion

In this prospective observational cohort study, we investigated the induced immune response in patients with infectious spondylodiscitis during and after antibiotic therapy. At baseline, the immune responses were either below or within the reference range. However, we found an increase in most of the induced immune responses as the patients were treated for infectious spondylodiscitis, and the overall cytokine release was within the reference range post-infection. Most of the unstimulated cytokine concentrations were higher at baseline than during antibiotic therapy, post-infection and when compared to references. No differences in the induced immune responses between patients with *S. aureus* versus Gram-negative bacteria, and patients with local disease versus disseminated disease were found.

Stimulation of peripheral blood mononuclear cells is a frequently used method for monitoring immune responses (11, 17, 18). However, immune responses to infections have high inter-individual variability (18). Standardized whole blood stimulation with TruCulture^®^ has proved to be highly reproducible and superior to conventional peripheral blood mononuclear cell stimulation for studying the immune responses (12, 18). In general, TruCulture^®^ has been used to investigate a range of pathologies in humans and animals including studies regarding patients with active infections. One study found that TruCulture^®^ was able to discriminate between active and latent tuberculosis by investigating stimulated IFN-γ responses (19). The TruCulture^®^ assay has also been used in several clinical studies at our center (13–16).

To our knowledge, this is the first study to investigate the induced immune response in patients with infectious spondylodiscitis using a standardized functional immune assay. A previous study on patients with infectious spondylodiscitis investigated the adaptive immune function by B- and T-lymphocyte phenotyping at diagnosis and three months after end of antibiotic therapy (10). The authors reported a profound and only partly reversible B-lymphocyte deficiency, and increased TCR-γδ T-lymphocytes, and proposed a possible immune dysfunction related to infectious spondylodiscitis. Furthermore, since adequate immune function is critical for the antibacterial host defense, we hypothesized that immunologic profiling with TruCulture^®^ would reveal an underlying immune deficiency in patients developing infectious spondylodiscitis. Importantly, this hypothesis was not supported, and our findings of the induced cytokine responses being within the reference range post-infection do not support the hypothesis of an underlying immune deficiency in patients with infectious spondylodiscitis.

We found a gradual increase in the induced immune responses from baseline to post-infection. This could indicate possible exhaustion of the immune system in patients with untreated infectious spondylodiscitis. Due to the non-specific nature of symptoms, diagnosis of infectious spondylodiscitis can be difficult, and a confirmatory diagnosis may be delayed (2, 8). In our study, the median time from onset of symptoms to diagnosis was 25 days (IQR 13-94) which could explain immune exhaustion. However, flow cytometric analyses were not performed. Thus, the present study cannot verify this hypothesis. Future studies on patients with infectious spondylodiscitis should combine functional assays with flow cytometry to investigate frequency and immune cell subsets as well as immune activation markers to determine possible immune exhaustion. Alternatively, an increase in induced immune responses from baseline to post-infection could be explained if the immune cells were concentrated at the sites where they are most active during the infection rather than in peripheral blood. Our results were based on blood samples solely and whether the cytokine concentrations in the tissue at site of inflammation were accurately reflected in the blood remains unclear. Thus, to investigate compartmentalization of the immune system in these patients, future studies should combine immunohistochemistry of tissue samples from site of inflammation and immune responses in analyses on blood samples.

*S. aureus* has evolved several mechanisms to modulate the innate and adaptive immune responses with potential severe infections as a result (20–23). This includes an ability of *S. aureus* to induce the host immune response to form abscesses, as frequently observed in patients with infectious spondylodiscitis, in favor of the pathogen to persist and induce productive inflammation (21). Additionally, mechanistic experiments on *S. aureus* suggests that human *S. aureus* isolates can produce several potent soluble factors ultimately causing lysis of innate immune cells, such as neutrophil granulocytes. Hypothetically, these characteristics could affect the immune system and therefore also the cytokine response differently from other bacteria in patients with infectious spondylodiscitis. This could potentially be reflected through immunologic profiling. Moreover, a previous study found that TruCulture^®^ could differentiate selected heat-killed commensal bacteria and highlighted a possibility of discriminating the causative pathogen in infections (24). However, we found no differences in the induced immune response in patients with infectious spondylodiscitis caused by *S. aureus* versus Gram-negative bacteria.

Furthermore, we found no evidence that TruCulture^®^ can be used as a predictor of disseminated disease in patients with infectious spondylodiscitis, and to our knowledge, TruCulture^®^ has not yet been used to predict dissemination of other bacterial infections. Thus, the findings of the present study do not support the implementation of TruCulture^®^ as a routine analysis for immune monitoring of patients with infectious spondylodiscitis. However, we acknowledge that due to the small study population, our results should be validated in larger cohorts.

Our study has some potential limitations. First, there was loss to follow-up. However, there was no difference in the immune function at baseline between patients with or without the post-infection blood sample (data not shown) and our results still consistently demonstrated a tendency of increased immune responses during treatment. Second, though our panel of designed stimulants covered important immunologic intra- and extracellular signaling pathways of the innate immune system, it lacked the ability to investigate other signaling pathways, including stimulation of the adaptive immune system. Third, the immune response was assessed by TruCulture^®^ alone, and combining TruCulture^®^ with other assays, such as flow cytometry, might improve the interpretation of the results and increase the quality of data. Fourth, the low number of study subjects, the variability in microbiological diagnoses, and the uneven distribution of disease manifestation may have prevailed us from finding differences within these subgroups, and our results should be validated in larger cohorts.

The strengths of this study include use of a standardized functional immune assay and the longitudinal design providing unique insight into the dynamics of induced immune responses in patients with infectious spondylodiscitis during and after a long-term and severe disease.

## Conclusion

This is the first study to investigate the induced immune response in patients with infectious spondylodiscitis using a standardized functional immune assay. We found lower induced immune responses in patients with infectious spondylodiscitis at baseline. However, we identified a general increase in the immune responses during treatment with normalized immune responses post-infection. Importantly, our results do not indicate an underlying immune deficiency in patients with infectious spondylodiscitis. We did not find evidence to support the use of induced immune responses as a tool for prediction of the causative pathogen or disease dissemination. Thus, our findings do not support implementation of TruCulture^®^ as a routine analysis in patients with infectious spondylodiscitis. Other methods should be explored to guide optimal treatment of patients with infectious spondylodiscitis.

## Data Availability Statement

The raw data supporting the conclusions of this article will be made available by the authors, without undue reservation.

## Ethics Statement

The studies involving human participants were reviewed and approved by The Research Ethics Committee of the Capital Region of Denmark (VEK) (H-17024315, approval number 79291), and The Danish Data Protection Agency (RH-2016-47, I-suite 04433). The patients/participants provided their written informed consent to participate in this study.

## Author Contributions

JL was responsible for concept, collection of the clinical data, statistical analyses and drafted the manuscript. DM and RT assisted in the statistical analyses and the draft of the manuscript. SO provided the immunologic data. SN was responsible for concept, data collection, supervision and edited the manuscript. All authors contributed to the design of the study and edited the manuscript. All authors contributed to the article and approved the submitted version.

## Funding

JL has received research grant from Rigshospitalets Forskningspuljer.

## Conflict of Interest

SN received an unrestricted research grant from the Novo Nordic Foundation.

The remaining authors declare that the research was conducted in the absence of any commercial or financial relationships that could be construed as a potential conflict of interest.

## Publisher’s Note

All claims expressed in this article are solely those of the authors and do not necessarily represent those of their affiliated organizations, or those of the publisher, the editors and the reviewers. Any product that may be evaluated in this article, or claim that may be made by its manufacturer, is not guaranteed or endorsed by the publisher.

## Abbreviations

ABT, Antibiotic therapy; Adj., Adjusted; *E. coli, Escherichia coli*; GN, Gram-negative; IL, Interleukin; INF, Interferon; IQR, Interquartile range; ISD, Infectious spondylodiscitis; LPS, Lipopolysaccharide; MRSA, Methicillin resistant *Staphylococcus aureus*; Poly:IC, Polyinosinic:polycytodylic acid; R848, Resiquimod; *S. aureus, Staphylococcus aureus*; TLR, Toll-Like receptor; TNF, Tumor necrosis factor.
