# Supplementary material for: The Induced Immune Response in Patients With Infectious Spondylodiscitis: A Prospective Observational Cohort Study
Source: Front Immunol. 2022 Mar 14;13:858934. doi: 10.3389/fimmu.2022.858934 (PMC8963848; doi:10.3389/fimmu.2022.858934)
Supplement: Supplementary file 1 [file Table_1.docx]

Supplementary Material

**Supplementary Table 1**: Cytokine concentrations at baseline and during antibiotic therapy.

| **LPS** | | | | | **R848** | | | | |
| --- | --- | --- | --- | --- | --- | --- | --- | --- | --- |
| **Cytokine** | **p-value** | **Adj. p-value** | **Baseline** | **During ABT** | **Cytokine** | **p-value** | **Adj. p-value** | **Baseline** | **During ABT** |
| **IFN-γ** | 0.0003  ** | 0.0021  * | 127.63 | 317.29 | **IFN-γ** | 0.0015  * | 0.0058  * | 226.98 | 418.93 |
| **IL-10** | 0.7696 | 0.7944 | 57.90 | 64.95 | **IL-10** | 0.4543 | 0.5591 | 267.60 | 262.0 |
| **IL-12** | 0.0505  * | 0.1243 | 1510.6 | 2265 | **IL-12** | 0.0003  ** | 0.0021  * | 1507.12 | 2633.2 |
| **IL-17A** | 0.6756 | 0.7721 | 20.40 | 22.86 | **IL-17A** | 0.7972 | 0.7972 | 11.59 | 10.624 |
| **IL-1b** | 0.0003  ** | 0.0021  * | 1938.43 | 3375 | **IL-1b** | 0.0010  ** | 0.0046  * | 957.35 | 1219.2 |
| **IL-6** | 0.2049 | 0.3278 | 7311.2 | 7471 | **IL-6** | 0.0505  * | 0.1243 | 4031 | 4482 |
| **IL-8** | 0.0283  * | 0.0822 | 5754.0 | 5403 | **IL-8** | 0.1338 | 0.2553 | 1606.2 | 1324.7 |
| **TNF-α** | 0.0016  * | 0.0058  * | 2431.3 | 4359.8 | **TNF-α** | 0.0002  ** | 0.0021  * | 2810.23 | 3523 |
| **Poly:IC** | | | | | **Unstimulated** | | | | |
| **Cytokine** | **p-value** | **Adj. p-value** | **Baseline** | **During ABT** | **Cytokine** | **p-value** | **Adj. p-value** | **Baseline** | **During ABT** |
| **IFN-γ** | 0.0825 | 0.1885 | 11.46 | 19.59 | **IFN-γ** | 0.2360 | 0.3596 | 0.01 | 0.74 |
| **IL-10** | 0.7513 | 0.7944 | 1.67 | 2.07 | **IL-10** | 0.65495 | 0.7698 | 1.53 | 1.98 |
| **IL-12** | 0.2804 | 0.4047 | 59.03 | 77.39 | **IL-12** | 0.2909 | 0.4047 | 0.01 | 82.08 |
| **IL-17A** | 0.1158 | 0.2470 | 1.84 | 0.01 | **IL-17A** | 0.1429 | 0.2553 | 1.51 | 0.01 |
| **IL-1b** | 0.7130 | 0.7868 | 1.20 | 1.31 | **IL-1b** | 0.3347 | 0.4463 | 0.73 | 0.73 |
| **IL-6** | 0.1436 | 0.2553 | 15.11 | 17.31 | **IL-6** | 2.784e-05  ** | 0.0009  ** | 4.81 | 1.95 |
| **IL-8** | 0.0100  * | 0.0321  * | 82.04 | 65.06 | **IL-8** | 0.4471 | 0.5591 | 44.27 | 32.87 |
| **TNF-α** | 0.0009  ** | 0.0046  * | 4.76 | 9.71 | **TNF-α** | 0.1953 | 0.3278 | 2.19 | 2.63 |

Abbreviations: ABT: antibiotic therapy. Adj., adjusted. IL, interleukin. IFN, interferon, LPS, lipopolysaccharide. Poly:IC, polyinosinic:polycytodylic acid. R848, Resiquimod. TNF, Tumor necrosis factor.

Table 3: The results were based on 43 patients with infectious spondylodiscitis, who contributed with a blood sample at baseline and during antibiotic therapy. The median concentration of each cytokine is represented, reported in pg/mL. Cytokine concentrations were compared using Exact Wilcoxon-Pratt Signed-Rank Test for paired, non parametric data. The adjusted p-value was the p-value after correction for multiple comparisons by the Benjamini-Hochberg procedure. (*p-value < 0.05,** p-value < 0.001)

**Supplementary Table 2:** Cytokine concentrations during antibiotic therapy and post-infection.

| **LPS** | | | | | **R848** | | | | |
| --- | --- | --- | --- | --- | --- | --- | --- | --- | --- |
| **Cytokine** | **p-value** | **Adj. p-value** | **During ABT** | **Post-infection** | **Cytokine** | **p-value** | **Adj. p-value** | **During ABT** | **Post-infection** |
| **IFN-γ** | 0.0728 | 0.1525 | 350.87 | 938.25 | **IFN-γ** | 0.6794 | 0.7765 | 726.92 | 854.89 |
| **IL-10** | 0.0446  * | 0.1097 | 63.45 | 87.39 | **IL-10** | 0.3955 | 0.5273 | 275.65 | 309 |
| **IL-12** | 0.0033  * | 0.0134  * | 2433.34 | 3741 | **IL-12** | 0.0874 | 0.1646 | 2904.94 | 4236 |
| **IL-17A** | 0.0062  * | 0.0220  * | 21.74 | 31.83 | **IL-17A** | 0.7983 | 0.8809 | 10.25 | 9.724 |
| **IL-1b** | 0.0028  * | 0.0130  * | 3908.64 | 8362 | **IL-1b** | 0.5412 | 0.6414 | 1608.36 | 1298 |
| **IL-6** | 0.0006  ** | 0.0051  * | 7752.27 | 10028 | **IL-6** | 0.4900 | 0.6031 | 4481.75 | 4456 |
| **IL-8** | 9.537e-05  ** | 0.0031  * | 5151.90 | 5718 | **IL-8** | 0.1336 | 0.2250 | 987.03 | 686 |
| **TNF-α** | 0.1819 | 0.2772 | 4790.30 | 6115 | **TNF-α** | 0.9217 | 0.9373 | 3877.27 | 3897 |
| **Poly:IC** | | | | | **Unstimulated** | | | | |
| **Cytokine** | **p-value** | **Adj. p-value** | **During ABT** | **Post-infection** | **Cytokine** | **p-value** | **Adj. p-value** | **During ABT** | **Post-infection** |
| **IFN-γ** | 0.0008  ** | 0.0051  * | 26.35 | 81.71 | **IFN-γ** | 0.9060 | 0.9373 | 2.27 | 3.17 |
| **IL-10** | 0.1083 | 0.1925 | 2.37 | 1.78 | **IL-10** | 0.0762 | 0.1525 | 2.53 | 1.40 |
| **IL-12** | 0.9373 | 0.9373 | 77.39 | 87.06 | **IL-12** | 0.4292 | 0.5494 | 41.41 | 10.79 |
| **IL-17A** | 0.1501 | 0.2402 | 1.61 | 1.33 | **IL-17A** | 0.0244  * | 0.0651 | 0.01 | 0.01 |
| **IL-1b** | 0.0088  * | 0.0282  * | 1.31 | 1.98 | **IL-1b** | 0.1978 | 0.2877 | 0.44 | 0.13 |
| **IL-6** | 0.0004  ** | 0.0051  * | 23.81 | 46.74 | **IL-6** | 0.0006  ** | 0.0051  * | 1.58 | 0.60 |
| **IL-8** | 0.2101 | 0.2923 | 49.82 | 29.98 | **IL-8** | 0.0728 | 0.1525 | 28.54 | 6.67 |
| **TNF-α** | 0.0181  * | 0.0526 | 11.27 | 13.85 | **TNF-α** | 0.0015  * | 0.0082  * | 1.66 | 1.02 |

Abbreviations: ABT: antibiotic therapy. Adj., adjusted. IL, interleukin. IFN, interferon, LPS, lipopolysaccharide. Poly:IC, polyinosinic:polycytodylic acid. R848, Resiquimod. TNF, Tumor necrosis factor.

Table 4: The results were based on 19 patients with infectious spondylodiscitis, who contributed with all three blood samples. The median concentration of each cytokine is represented, reported in pg/mL. Cytokine concentrations were compared using Exact Wilcoxon-Pratt Signed-Rank Test for paired, non parametric data. The adjusted p-value was the p-value after correction for multiple comparisons by the Benjamini-Hochberg procedure. (*p-value < 0.05,** p-value < 0.001)

**Supplementary Table 3**: Cytokine concentrations in patients with infectious spondylodiscitis caused by *S. aureus* and Gram-negative bacteria.

| **LPS** | | | | | **R848** | | | | |
| --- | --- | --- | --- | --- | --- | --- | --- | --- | --- |
| **Cytokine** | **p-value** | **Adj. p-value** | ***S. aureus*** | **GN-bacteria** | **Cytokine** | **p-value** | **Adj. p-value** | ***S. aureus*** | **GN-bacteria** |
| **IFN-γ** | 0.9564 | 1.0000 | 64.90 | 119 | **IFN-γ** | 0.2781 | 0.6844 | 227 | 77 |
| **IL-10** | 0.2863 | 0.6844 | 75.41 | 46.3 | **IL-10** | 1.00 | 1.0000 | 260.9 | 251.5 |
| **IL-12** | 0.6088 | 0.8470 | 1160.1 | 1040.4 | **IL-12** | 0.8115 | 0.9390 | 1002.7 | 1039.0 |
| **IL-17A** | 0.6088 | 0.8470 | 20.40 | 22.5 | **IL-17A** | 0.3193 | 0.6844 | 18.2 | 15.6 |
| **IL-1b** | 0.073 | 0.6292 | 1697.5 | 646.9 | **IL-1b** | 0.0243  * | 0.6292 | 1169.4 | 464.6 |
| **IL-6** | 0.1351 | 0.6292 | 7324.5 | 5660 | **IL-6** | 0.3917 | 0.7373 | 4581 | 3048 |
| **IL-8** | 0.2273 | 0.6844 | 6245 | 5477.9 | **IL-8** | 0.0865 | 0.6292 | 1890.5 | 1385.9 |
| **TNF-α** | 0.3917 | 0.7373 | 1961.6 | 1163.5 | **TNF-α** | 0.7077 | 0.8710 | 2438.2 | 2120.7 |
| **Poly:IC** | | | | | **Unstimulated** | | | | |
| **Cytokine** | **p-value** | **Adj. p-value** | ***S. aureus*** | **GN-bacteria** | **Cytokine** | **p-value** | **Adj. p-value** | ***S. aureus*** | **GN-bacteria** |
| **IFN-γ** | 0.6989 | 0.8710 | 2.40 | 1.87 | **IFN-γ** | 0.1499 | 0.6292 | 0.01 | 0.01 |
| **IL-10** | 0.1573 | 0.6292 | 0.64 | 2.78 | **IL-10** | 0.1118 | 0.6292 | 1.11 | 2.9 |
| **IL-12** | 0.9814 | 1.0000 | 75.03 | 56.38 | **IL-12** | 0.8555 | 0.9440 | 0.01 | 0.01 |
| **IL-17A** | 0.0873 | 0.6292 | 1.83 | 7.35 | **IL-17A** | 0.8216 | 0.9390 | 0.44 | 2.78 |
| **IL-1b** | 0.3208 | 0.6844 | 1.78 | 0.94 | **IL-1b** | 0.5078 | 0.7738 | 1.20 | 0.44 |
| **IL-6** | 0.6576 | 0.8710 | 14.89 | 9.31 | **IL-6** | 0.5069 | 0.7738 | 7.01 | 9.63 |
| **IL-8** | 0.4727 | 0.7738 | 100.60 | 102.56 | **IL-8** | 0.2557 | 0.6844 | 63.76 | 46.94 |
| **TNF-α** | 0.2322 | 0.6844 | 3.85 | 2.32 | **TNF-α** | 0.5047 | 0.7738 | 2.35 | 1.42 |

Abbreviations: Adj., adjusted. GN, Gram-negative. IL, interleukin. IFN, interferon, LPS, lipopolysaccharide. Poly:IC, polyinosinic:polycytodylic acid. R848, Resiquimod. *S. aureus, Staphylococcus aureus.* TNF, Tumor necrosis factor.

Table 5: Seventeen of the patients had infectious spondylodiscitis caused by *S. aureus*, and six of the patients had infectious spondylodiscitis caused by Gram-negative bacteria. The median concentration of each cytokine is represented, reported in pg/mL. Cytokine concentrations were compared using Wilcoxon-Mann-Whitney Test for unpaired, non parametric data. The adjusted p-value was the p-value after correction for multiple comparisons by the Benjamini-Hochberg procedure. (*p-value < 0.05,** p-value < 0.001).

**Supplementary Table 4:** Cytokine concentrations in patients with infectious spondylodiscitis with local disease and disseminated disease.

| **LPS** | | | | | **R848** | | | | |
| --- | --- | --- | --- | --- | --- | --- | --- | --- | --- |
| **Cytokine** | **p-value** | **Adj. p-value** | **Disseminated**  **ISD** | **Local ISD** | **Cytokine** | **p-value** | **Adj. p-value** | **Disseminated**  **ISD** | **Local ISD** |
| **IFN-γ** | 0.1392 | 0.5949 | 103.2 | 184.6 | **IFN-γ** | 0.8527 | 0.8802 | 245.8 | 145.1 |
| **IL-10** | 0.7121 | 0.8138 | 57.7 | 91.4 | **IL-10** | 0.1275 | 0.5949 | 278.4 | 229.6 |
| **IL-12** | 0.1714 | 0.5949 | 1361.7 | 2661.4 | **IL-12** | 0.7087 | 0.8138 | 1495.9 | 1462.4 |
| **IL-17A** | 0.4768 | 0.6545 | 23.5 | 19.7 | **IL-17A** | 0.0345  * | 0.5949 | 16.8 | 8.8 |
| **IL-1b** | 0.3069 | 0.6356 | 1963.0 | 3046 | **IL-1b** | 0.4004 | 0.6545 | 1037.4 | 927.3 |
| **IL-6** | 0.1801 | 0.5949 | 7296.8 | 8820 | **IL-6** | 0.2117 | 0.5949 | 4529 | 3918.8 |
| **IL-8** | 0.6304 | 0.7759 | 5676.3 | 5903 | **IL-8** | 0.2201 | 0.5949 | 1824.4 | 1288.6 |
| **TNF-α** | 0.2859 | 0.6356 | 2393.7 | 3368 | **TNF-α** | 0.4804 | 0.6545 | 2839.1 | 2197.3 |
| **Poly:IC** | | | | | **Unstimulated** | | | | |
| **Cytokine** | **p-value** | **Adj. p-value** | **Disseminated**  **ISD** | **Local ISD** | **Cytokine** | **p-value** | **Adj. p-value** | **Disseminated**  **ISD** | **Local ISD** |
| **IFN-γ** | 0.0763 | 0.5949 | 7.95 | 13.66 | **IFN-γ** | 0.4214 | 0.6545 | 0.01 | 0.01 |
| **IL-10** | 0.2231 | 0.5949 | 1.65 | 0.54 | **IL-10** | 0.271 | 0.6356 | 1.46 | 1.55 |
| **IL-12** | 0.8403 | 0.8802 | 68.81 | 59.03 | **IL-12** | 0.4741 | 0.6545 | 15.67 | 0.01 |
| **IL-17A** | 0.1021 | 0.5949 | 2.93 | 1.53 | **IL-17A** | 0.6174 | 0.7759 | 1.67 | 1.53 |
| **IL-1b** | 0.7803 | 0.8610 | 1.00 | 1.78 | **IL-1b** | 0.9584 | 0.9584 | 0.78 | 0.74 |
| **IL-6** | 0.3517 | 0.6545 | 14.81 | 16.26 | **IL-6** | 0.0420  * | 0.5949 | 5.31 | 3.00 |
| **IL-8** | 0.3178 | 0.6356 | 95.01 | 54.77 | **IL-8** | 0.0645 | 0.5949 | 59.16 | 31.06 |
| **TNF-α** | 0.4035 | 0.6545 | 4.68 | 6.54 | **TNF-α** | 0.4909 | 0.6465 | 2.21 | 1.97 |

Abbreviations: Abbreviations: Adj., adjusted. IL, interleukin. IFN, interferon. ISD, infectious spondylodiscitis. LPS, lipopolysaccharide. Poly:IC, polyinosinic:polycytodylic acid. R848, Resiquimod. TNF, Tumor necrosis factor.

Table 6: Thirty-six of the patients with infectious spondylodiscitis had disseminated disease and 13 of the patients had local disease. The median concentration of each cytokine is represented, reported in pg/mL Cytokine concentrations were compared using Wilcoxon-Mann-Whitney Test for unpaired, non parametric data. The adjusted p-value was the p-value after correction for multiple comparisons by the Benjamini-Hochberg procedure. (*p-value < 0.05,** p-value < 0.001).
